# Symptomatic Meckel’s Diverticulum Presenting As Recurrent Gastrointestinal Bleeding in an Adult

**DOI:** 10.7759/cureus.41723

**Published:** 2023-07-11

**Authors:** Abishek Hariharan, Shefali Amin, Salina Munankami, Manish Shrestha, John Altomare

**Affiliations:** 1 Internal Medicine, Tower Health Medical Group, West Reading, USA; 2 General Medicine, Kathmandu Medical College, Kathmandu, NPL; 3 Gastroenterology, Tower Health Medical Group, West Reading, USA

**Keywords:** esophagogastroduodenoscopy, hematochezia, obscure gi bleed, video capsule, colonoscopy, meckel’s scan, acute blood loss anemia, meckel´s diverticulum

## Abstract

A 51-year-old male presented to the hospital with recurrent gastrointestinal bleeding. Prior work up with an esophagogastroduodenoscopy (EGD), colonoscopy, and video capsule endoscopy failed to reveal a bleeding source. Given a history of a terminal ileum diverticulum noted on previous colonoscopy and persistence of hematochezia, a Meckel’s scan was performed, which revealed abnormal uptake suspicious for a Meckel’s diverticulum containing ectopic gastric mucosa. After surgical resection, pathology confirmed a Meckel's diverticulum with gastric heterotopia. This case highlights the importance of considering Meckel’s diverticulum for instances of recurrent gastrointestinal bleeding, especially in patients who are still symptomatic despite an extensive workup. Moreover, it is important to note that a Meckel's diverticulum can be missed on video capsule endoscopy.

## Introduction

Meckel’s diverticulum is a congenital gastrointestinal malformation due to the incomplete obliteration of the vitelline duct resulting in a true diverticulum of the small intestinal as it contains all three layers of the small bowel wall [1]. While it is often asymptomatic, it can occasionally present with abdominal pain, gastrointestinal bleeding, or bowel obstruction symptoms [1,2]. More commonly seen within the pediatric population, it can sometimes be present among adults [1]. In this report, we discuss a case of Meckel’s diverticulum in a 51-year-old male with recurrent gastrointestinal bleeding episodes who was ultimately diagnosed via a Meckel’s scan and subsequently confirmed with pathology following surgical resection.

This article was previously presented as a meeting abstract at the 2022 American College of Gastroenterology (ACG) Annual Scientific Meeting on October 24, 2022. 

## Case presentation

A 51-year-old male with a past medical history of iron deficiency anemia, hypothyroidism, posttraumatic stress disorder, insomnia, and depression presented to the hospital complaining of bloody stools. One year prior, he had a similar presentation that was evaluated with esophagogastroduodenoscopy (EGD) and colonoscopy. EGD and colonoscopy revealed mild gastritis, mild sigmoid diverticulosis, and fresh blood within the terminal ileum but no source of bleeding. Video capsule endoscopy was also deployed and showed fresh bleeding within the terminal ileum just proximal to the ileocecal valve but failed to reveal a bleeding source. A repeat colonoscopy six months later revealed sigmoid diverticulosis and a single non-bleeding diverticulum in the terminal ileum; however, no fresh blood or source of bleed was identified. An outpatient double-balloon enteroscopy was recommended. Unfortunately, it was not able to be completed due to this current hospitalization. The patient presented to the hospital after five episodes of acute bright red blood per rectum. He presented as afebrile with a heart rate of 128 bpm and blood pressure of 93/58 mmHg. Review of systems was positive for diaphoresis, lightheadedness, nausea, pale skin, and mild epigastric tenderness. The patient denied shortness of breath, chest pain, hematuria, non-steroid anti-inflammatory drugs (NSAID) use, chronic anticoagulation, alcohol use, or tobacco use. Initial laboratory studies (Table 1) were remarkable for acute on chronic anemia. 

**Table 1:** Laboratory Values on Admission vs Six Months Prior All relevant laboratory values are shown in the table. Other lab values that are part of the basic work up are not shown here, but were within normal limits.

Laboratory Test	Lab Value on Admission	Lab Value Six Months Prior	Reference Range
White blood cell count (x10^3^/µl)	9.5	5.2	4.8-10.8
Hemoglobin (g/dL)	10.6	11.4	12-16
Hematocrit (%)	33	38.9	39-53
Platelet count (x10^3^/µl)	276	351	130-400
Mean corpuscular volume (fL)	78.9	71.9	80-99
Blood urea nitrogen (mg/dL)	27	19	7-25
International normalized ratio (INR)	1.1	-	0.9-1.1

**Figure 1:**
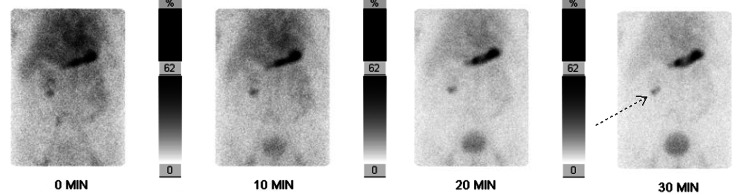
Meckel’s Scan Revealing Abnormal Uptake (Arrow) in the Right Mid to Lower Abdomen

Abdominal physical examination was unremarkable: normal bowel sounds, non-tender to light and deep palpation, and a non-distended abdomen. CT scan of the abdomen and pelvis with contrast showed no signs of active gastrointestinal bleeding. The patient was managed with intravenous fluid resuscitation, continuous pantoprazole infusion, one unit of packed red blood cells, and made nil per os (NPO) until evaluation by the gastroenterology team. The patient underwent repeat EGD and colonoscopy, which were unremarkable except for blood-tinged fluid interspersed throughout the colon without active hemorrhage. The source of bleeding remained obscure. Given the history of a terminal ileum diverticulum and persistence of hematochezia, a Meckel's scan was performed (Figure 1), revealing abnormal uptake in the right mid to lower abdomen suspicious for a Meckel's diverticulum containing ectopic gastric mucosa. The patient was taken to the operating room the same day, and a large Meckel's diverticulum was noted at approximately two feet proximal to the ileocecal valve, along with hemorrhagic inflammatory changes within the local mesentery. He underwent a partial small bowel resection and an appendectomy with pathology revealing a Meckel's diverticulum of the ileum with gastric heterotopia. The patient had an uncomplicated recovery postoperatively, along with the resolution of his hematochezia.

## Discussion

Meckel's diverticulum is a persistent remnant of the omphalomesenteric duct that ultimately fails to involute by the fifth and sixth week of gestation. The failure of the duct to involute can result in various anatomic patterns, including the formation of omphalomesenteric cysts, fistulas, fibrous bands, and most commonly, a diverticulum that can persist into adulthood [3].

It is difficult to determine the incidence of Meckel's diverticulum as it is often asymptomatic, with no simple screening studies that can identify its presence. It is, in fact, more likely to be discovered incidentally during an abdominal surgical exploration for an unrelated cause [2,3]. However, based on various prospective studies analyzed, the incidence is between 0.3-2.9% [2]. Symptoms are usually nonspecific and can include gastrointestinal bleeding, signs of obstruction, and inflammation. Bleeding is generally associated with ectopic gastric mucosa seen within the diverticula, which produces gastric secretions that cause mucosal ulceration of the adjacent small bowel [2]. Symptoms may also present in the form of bowel obstruction secondary to forming a volvulus, intussusception, diverticular inflammation, herniation, or perforation, with the diverticula acting as a lead point from which the obstruction occurs [2]. Acute abdominal pain may also be present, often mimicking symptoms of acute appendicitis. Suspicion of Meckel's diverticulum should be high if signs of appendicitis are present in patients who have previously undergone an appendectomy [2,3]. Abdominal pain occurs from underlying inflammation within the diverticular pocket that can increase the risk of perforation and even ischemia in rare occurrences [4]. There are reports of tumor formation from Meckel's diverticulum, with an incidence rate of 0.5-3.2%; however, these are often benign [4].

Physical examination in a patient with Meckel's diverticulum is often nonspecific, and laboratory studies may reveal signs of volume depletion or anemia [5]. Unfortunately, due to its relatively rare prevalence and nonspecific symptoms, other sources of abdominal pain and bleeding are often first considered in these patients, making it a diagnostic challenge [5]. Furthermore, imaging studies such as an ultrasound, abdominal x-ray, and CT have limited diagnostic value in detecting Meckel's diverticulum [5,6]. In spite of its challenging presentation, there should be a suspicion for Meckel's diverticulum in adult patients who present with recurrent gastrointestinal bleeding without an obvious source of bleed seen in endoscopy or colonoscopy. In these patients, a wireless capsule endoscopy is usually performed to determine the source of the bleed of uncertain origin [3,5,6]. The test has a variable sensitivity ranging from 81.8-84.6% to detect a bleeding Meckel's diverticulum, making it a valuable but unreliable diagnosis tool [5,6].

Meanwhile, the technetium-99m pertechnetate scan, also known as the Meckel's scan, is a nonspecific test for Meckel's diverticulum, which can be used due to its ability to detect the presence of ectopic gastric mucosal tissue [2,5]. However, this comes with the drawback of poor sensitivity (60%) in the adult population relative to the pediatric population (85-97%) and a decreased likelihood of yielding a positive result in cases without ectopic tissue within the diverticula [6,7]. There have also been reports of the use of double-balloon enteroscopy when the suspicion for Meckel's diverticulum remains high given its improved visualization of the entire small bowel [3,8]. Yet, it comes at the cost of prolonged sedation and elevated risk of pancreatitis relative to a standard endoscopy [8]. Alternative options include conventional contrast mesenteric arteriography for brisk bleeds and a high-resolution CT angiography to detect active bleeds as slow as 0.3 mL/minute [9]. However, neither test is diagnostic for Meckel's diverticulum [8,9].

In patients with Meckel's diverticulum, initial management is directed toward stabilizing a patient's hypovolemia, anemia, or symptoms of obstruction. Following the eventual diagnosis of Meckel's diverticulum, most symptomatic patients will require surgical resection of the diverticula via diverticulectomy or small bowel resection and primary anastomosis [2,3]. In cases with asymptomatic Meckel's diverticulum, the data is currently unclear and is at the discretion of the provider [3]. Surgery is not routinely performed when diagnosed incidentally via imaging on asymptomatic patients; however, there continues to be debate regarding the criteria for surgery for asymptomatic patients [10]. One study recommends a Risk Score based on four risk factors: male sex, patients below 45 years of age, diverticula longer than 2 cm, and fibrous bands [2,10]. Resection in an asymptomatic patient may be performed if the risk score is greater than or equal to 6 [3,10].

## Conclusions

This case demonstrates the presence of Meckel’s diverticulum in an adult male that was not diagnosed despite multiple episodes of lower gastrointestinal bleeding and an extensive workup. A video capsule study, while a very sensitive study, may not always detect a Meckel's diverticulum, especially if an active bleed is not present. In such scenarios, a Meckel's scan would be beneficial to make the final diagnosis. Despite its rare occurrence, Meckel's diverticulum should be considered in cases of gastrointestinal bleeding without an apparent source.
